# Invasive *Aedes* mosquitoes in an urban—peri-urban gradient in northern Spain: evidence of the wide distribution of *Aedes japonicus*

**DOI:** 10.1186/s13071-023-05862-6

**Published:** 2023-07-14

**Authors:** Aitor Cevidanes, Fátima Goiri, Jesús F. Barandika, Patricia Vázquez, Joseba Goikolea, Ander Zuazo, Natalia Etxarri, Gurutze Ocio, Ana L. García-Pérez

**Affiliations:** 1 Animal Health Department, NEIKER-Basque Institute for Agricultural Research and Development, Basque Research and Technology Alliance (BRTA), Derio, Bizkaia Spain; 2 Subdirección de Salud Pública de Gipuzkoa, Eusko Jaurlaritza-Gobierno Vasco, Donostia, Gipuzkoa Spain; 3 Dirección de Sanidad Ambiental e Higiene Urbana, Área de Salud y Consumo del Ayuntamiento de Bilbao, Bilbao, Bizkaia Spain; 4 Dirección de Medio Ambiente, Sección de Sanidad Alimentaria y Zoonosis del Ayuntamiento de Donostia, Donostia, Gipuzkoa Spain; 5 Departamento de Deporte y Salud, Servicio de Salud Pública, Unidad Sanitaria de Consumo del Ayuntamiento de Vitoria-Gasteiz, Vitoria-Gasteiz, Araba Spain

**Keywords:** *Aedes albopictus*, *Aedes japonicus*, Invasive mosquitoes, Urbanization, Entomological surveillance, Ovitraps, COI gene

## Abstract

**Background:**

The expansion of invasive mosquitoes throughout Europe has increased in recent decades. In northern Spain, *Aedes albopictus* was detected for the first time in 2014, and *Aedes japonicus* was detected in the three Basque provinces in 2020. This study aimed to evaluate the distribution of these mosquito species and their association with factors related to urbanization.

**Methods:**

In 2021, a total of 568 ovitraps were deployed in 113 sampling sites from 45 municipalities with > 10,000 inhabitants. Oviposition substrate sticks were replaced each fortnight and examined for *Aedes* eggs from June to November. *Aedes* eggs were counted, and the eggs from a selection of positive oviposition sticks, encompassing at least one stick from each positive ovitrap, were hatched following their life cycle until the adult stage. When egg hatching was not successful, PCR targeting the COI gene and sequencing of amplicons were carried out.

**Results:**

Eggs were detected in 66.4% of the sampling sites and in 32.4% of the ovitraps distributed in the three provinces of the Basque Country. *Aedes albopictus* and *Ae. japonicus* were widespread in the studied area, confirming their presence in 23 and 26 municipalities, respectively. Co-occurrence of both species was observed in 11 municipalities. The analysis of the presence of *Aedes* invasive mosquitoes and the degree of urbanization (urban, suburban, peri-urban) revealed that *Ae. albopictus* showed a 4.39 times higher probability of being found in suburban areas than in peri-urban areas, whereas *Ae. japonicus* had a higher probability of being found in peri-urban areas. Moreover, the presence of *Ae. albopictus* was significantly associated with municipalities with a higher population density (mean = 2983 inh/km^2^), whereas *Ae. japonicus* was associated with lower population density (mean = 1590 inh/km^2^).

**Conclusions:**

The wide distribution of *Ae. albopictus* and *Ae. japonicus* observed confirmed the spread and establishment of these species in northern Spain. A new colonization area of *Ae. japonicus* in Europe was confirmed. Due to the potential impact of *Aedes* invasive mosquitoes on public health and according to our results, surveillance programs and control plans should be designed considering different urbanization gradients, types of environments, and population density.

**Graphical Abstract:**

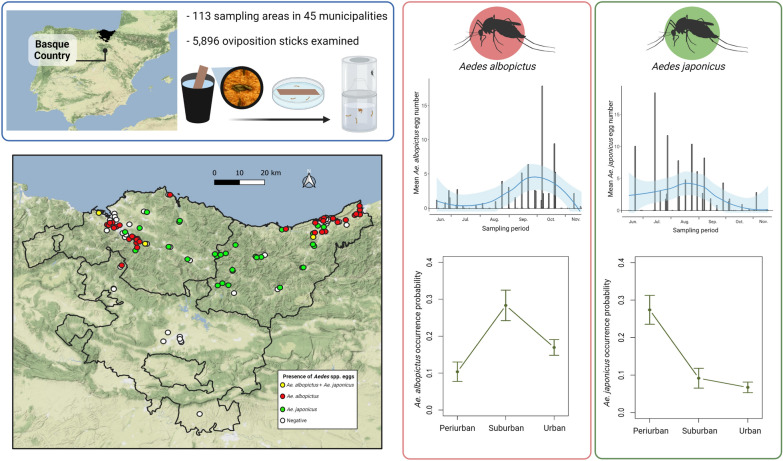

## Background

Mosquitoes are of paramount significance in terms of animal and public health due to their role as vectors of several pathogens and because of the impact they have as biting and annoying insects. Globalization, human travel, and global trade have facilitated the spread of exotic invasive mosquitoes of the genus *Aedes* [1]. Additionally, climatic and environmental changes increase the possibility of the spread and establishment of some species in new regions. In Europe, six species of *Aedes* invasive mosquitoes (AIM) (*Aedes albopictus*, *Aedes aegypti*, *Aedes japonicus*, *Aedes koreicus, Aedes atropalpus*, and *Aedes triseriatus*) have been introduced since the 1970s [2], and some have known established populations [3, 4]. Among them, only two species are present in Spain [2].

*Aedes albopictus* (Skuse, 1894) is an exotic invasive species that was introduced into Albania during the late 1970s and later in Italy in 1990 [5, 6] and is currently widely distributed in Europe. Its ability to adapt to cold temperatures and overwinter in temperate regions and its plasticity to adapt to different habitats, together with globalization and climate change, contribute to the successful invasion of this multivoltine species [7]. Urban emplacements provide suitable habitats and resources for *Ae. albopictus* development, allowing them to colonize and proliferate; it is considered a container-breeding mosquito, and its ability to occupy habitats in urban environments is noticeable. This species represents a public health concern since it can act as a vector of some arboviruses, most notably dengue, chikungunya, and Zika [7]. In fact, several autochthonous outbreaks have been recently reported in Europe, thus confirming local transmission of these diseases in places where *Ae. albopictus* has been established [8–12]. The epidemiology of vector-borne pathogen transmission is driven by the interaction among vector, host, and pathogen and is profoundly affected by urbanization processes. As urbanization processes increase globally, many countries are experiencing the re-emergence and introduction of vector-borne diseases [13]. In addition, the aggressive biting behavior of *Ae. albopictus* jeopardizes the quality of life of local citizens and can even have a direct impact on the regional economy. In Spain, after the first detection of *Ae. albopictus* in Catalonia in 2004, the Spanish Ministry of Health launched a surveillance campaign in 2007 in several Mediterranean Spanish regions [14]. In northern Spain, *Ae. albopictus* was identified for the first time in 2014 in the Basque Country, on the border with France, and since then, it has been expanding toward new areas [15].

*Aedes japonicus* (Theobald, 1901) was first detected in 2000 in France in a storage yard of imported tires [16]. Since then, this species has been reported mostly in central Europe [17, 18]. *Aedes japonicus* is well adapted to temperate climates and is capable of withstanding cold and snowy winters. It is a multivoltine species, and larval hatching occurs early in the year as soon as breeding sites lose their ice cover at water temperatures of 4.0–4.5 °C [17]. This species has an adaptive capacity, and they have been observed to displace other indigenous mosquito species [19]. The activity period of adult mosquitoes can last until early December. In contrast to *Ae. albopictus*, this species prefers forested and bushy areas, and larvae can be found in tree holes, tree stumps, rainwater pools [17], and other natural or artificial containers. *Aedes japonicus* is a potential vector of several viruses of medical and veterinary importance, such as chikungunya, dengue, Zika, and West Nile [20]. This species is not considered a primary nuisance species to humans [21], but it is of particular concern because of its ability to rapidly adapt to new habitats given its high tolerance to a broad range of climate conditions [19, 22]. *Aedes japonicus* was detected in northern Spain (Asturias) in 2018 through the citizen science platform Mosquito Alert [23] and 2 years later was identified in two close regions (Cantabria and the Basque Country) [2, 24], suggesting a new colonization area in Europe.

Urbanization processes have a major impact on mosquito communities by decreasing species richness and increasing the abundance of selected mosquito species such as *Ae. albopictus*, which are very well adapted to urban and suburban ecosystems [13, 25]. Until now, during the surveillance program carried out in the Basque Country, the selection of sampling areas was focused on places with heavy road traffic (such as car parks, petrol stations, or logistics platforms, among others) to increase the probability of detecting *Ae. albopictus* [15]. However, recent findings [2, 24] lead us to think that *Ae. japonicus* could also be established in this area. Thus, considering that *Ae. japonicus* inhabits areas with abundant vegetation, being more abundant in rural settings [25], this study focuses on (i) evaluating the association of the presence of AIM with factors related to the type of environment, urbanization degree and population density and (ii) investigating the range of distribution of *Ae. japonicus* in the Basque country.

## Methods

### Study area

The Basque Country is a small region in northern Spain with ca. 7200 km^2^ divided into three administrative provinces: Gipuzkoa, Bizkaia, and Araba. The average annual temperature and rainfall are 13.4 °C and 1610 mm in Gipuzkoa, 13.8 °C and 1278 mm in Bizkaia, and 11.5 °C and 878 mm in Araba. The population of the Basque Country is *ca.* 2,188,017 inhabitants, and industry and tourism are some of the driving forces of the Basque economy [26]. There is a network of highly trafficked motorways structured around the main routes that connect the Basque Country with France, central Spain, and the Mediterranean coast, facilitating the introduction of AIM to new areas by passive transportation.

### Sampling approach

The sampling strategy to monitor AIM presence in 2021 encompassed the placement of ovitraps in 45 municipalities, most of them with > 10,000 inhabitants. Two sampling zones were selected in each municipality, with the exception of the three main cities (Bilbao, Donostia/San Sebastian, and Vitoria-Gasteiz), where the number of selected zones increased to 8, 17, and 4, respectively. A total of 113 sampling areas were selected.

Fieldwork was carried out by a network of health officers from the Public Health Directorate of the Basque Government and from the municipalities of Bilbao, Donostia/San Sebastian, Vitoria-Gasteiz, and Laguardia. Sampling started on June 1 and finished on November 18. Thus, each municipality and area was sampled from 11 to 12 times over a period of 23 weeks (June–November).

### Sampling methodology

The presence of *Aedes* spp. eggs was investigated using oviposition traps (ovitraps) [27]. A total of 568 ovitraps were deployed (Gipuzkoa: 275 ovitraps, 19 municipalities, 55 sampling areas; Bizkaia: 235 ovitraps, 22 municipalities, 47 sampling areas; Araba: 58 ovitraps, 4 municipalities, 11 sampling areas) (Table 1). Five ovitraps were placed in each sampling area, always in shady places and hidden in the vegetation. Each ovitrap contained dechlorinated water and a wooden stick (masonite; 12 cm long and 2.5 cm width) as oviposition substrate. Oviposition sticks were removed and replaced by new sticks every 15 days.

**Table 1:** Number of municipalities, sampling areas, ovitraps, and oviposition sticks examined in the three Basque provinces and prevalence of eggs of *Aedes* spp.

Province	No. municipalities	Sampling areas		Ovitraps		Oviposition sticks examined	
		No.	Posit. *Aedes* eggs (%)	No.	Posit. *Aedes* eggs (%)	No.	Posit. *Aedes* eggs (%)
Araba	4	11	1 (9.1)	58	2 (3.4)	559	2 (0.4)
Bizkaia	22	47	31 (66.0)	235	65 (27.7)	2484	124 (5.0)
Gipuzkoa	19	55	43 (78.2)	275	117 (42.5)	2853	349 (12.2)
Total	45	113	75 (66.4)	568	184 (32.4)	5896	475 (8.1)

Information on each sampling area was compiled, including geographic coordinates and type of environment (parking, green park, petrol station, city center, industrial zone). Sampling sites were also categorized according to the urbanization degree: urban, suburban, and peri-urban areas. Based on Loibl et al. [28] and adjusted to the socio-geographical reality of the region, the sampling areas were categorized as follows: Urban areas were those in the town center, including urban core and inner urban areas; suburban areas were mainly residential, not densely compacted, and located near an inner urban area; peri-urban areas were spaces located on the boundaries of the town with scarce urban development, and including both urban-fringe and urban periphery.

### Laboratorial methods

Oviposition sticks were examined in the laboratory under a stereomicroscope (zoom magnification range of 7.5–135 ×). If eggs were observed, they were counted, and positive sticks were preserved for egg hatching by immersion in a petri dish with dechlorinated water. Emerged larvae were placed in mosquito breeders (Bioquip^®^, Compton, CA, USA) at room temperature (ca. 23 °C) until becoming adult mosquitoes. The identification of *Aedes* mosquitoes was performed using taxonomic keys [29]. In the case of unsuccessful egg hatching, 5–10 eggs were collected from selected sticks, and DNA extraction was performed using a commercial kit (NZY Tissue gDNA isolation kit, NZYtech, Lisboa, Portugal), followed by PCR targeting the cytochrome c oxidase I subunit (COI) gene [30]. Amplicons were sequenced by external services using the Sanger technique. Sequences were compared with those available in GenBank by BLAST analysis to confirm the species. A selection of sequences is deposited in GenBank with the reference numbers OQ884140-OQ884148.

### Statistical analyses

The positive ovitrap index (POI) [(number of positive traps/number of inspected traps) × 100] was estimated for each sampling area [31]. A sampling area was considered positive when the presence of *Aedes* spp. eggs was detected in at least one oviposition trap in at least one sampling. To represent the overall egg-laying kinetics of AIMs over the sampling period (week 24–week 46), the means of eggs counted each fortnight were calculated and represented in a graph with a smoothed trend line based on LOESS (locally estimated scatterplot smoothing). Associations between the presence of *Ae. albopictus* and *Ae. japonicus* (as a dependent binomial variable) with province, urbanization (urban/suburban/peri-urban) and type of environment (town, petrol station, parking, green parks and industrial areas) were analyzed using logistic regression models (GLM), and odds ratios (OR) were calculated using the ORci function of the CIplot package. Censuses from each municipality included in the study were compiled. Thus, the presence of each species according to the human population density of the municipality (inh/km^2^) was evaluated by the Wilcoxon rank-sum test. All statistical analyses were performed using R statistical software version 3.6.1 [32].

## Results

### Distribution of invasive mosquitoes

A total of 5896 oviposition sticks were examined in 2021 (2853 from Gipuzkoa, 2484 from Bizkaia; 559 from Araba). Eggs of *Aedes* spp. were detected in 66.4% of the sampling sites, and 32.4% of the ovitraps were distributed in the three territories of the Basque Country (Table 1). In 18 sampling sites from 13 municipalities, only one positive ovitrap and one oviposition stick were positive throughout the surveillance period. In contrast, in 10 sampling sites from 6 municipalities, the five ovitraps harbored *Aedes* eggs during more than 8 samplings. The province of Gipuzkoa showed a higher percentage of positive ovitraps (42.5%) than Bizkaia (27.7%) and Araba (3.4%). Likewise, the percentage of oviposition sticks with eggs was higher in Gipuzkoa (12.2% vs. 5.0% in Bizkaia and 0.4% in Araba) (Table 1).

Notably, 100% of the municipalities in Gipuzkoa were positive for the presence of *Aedes* spp., while in Araba, *Aedes* eggs were detected only in one of the four municipalities investigated (Table 2).

**Table 2:** Municipalities included in the surveillance programme and the number of them harboring AIM, *Aedes albopictus, Ae. japonicus*, or both species simultaneously

Province	No. municipalities	Total with AIM (% pos)	*Ae. albopictus* (% pos)	*Ae. japonicus* (% pos)	*Ae. albopictus* and *Ae. japonicus* (% pos)
Araba	4	1 (25.0)	0	0	1 (25.0)
Bizkaia	22	18 (81.8)	6 (27.3)	5 (22.7)	7 (31.8)
Gipuzkoa	19	19 (100)	6 (31.6)	10 (52.6)	3 (15.8)
Total	45	38 (84.4)	12 (26.7)	15 (33.3)	11 (24.4)

**Fig. 1:**
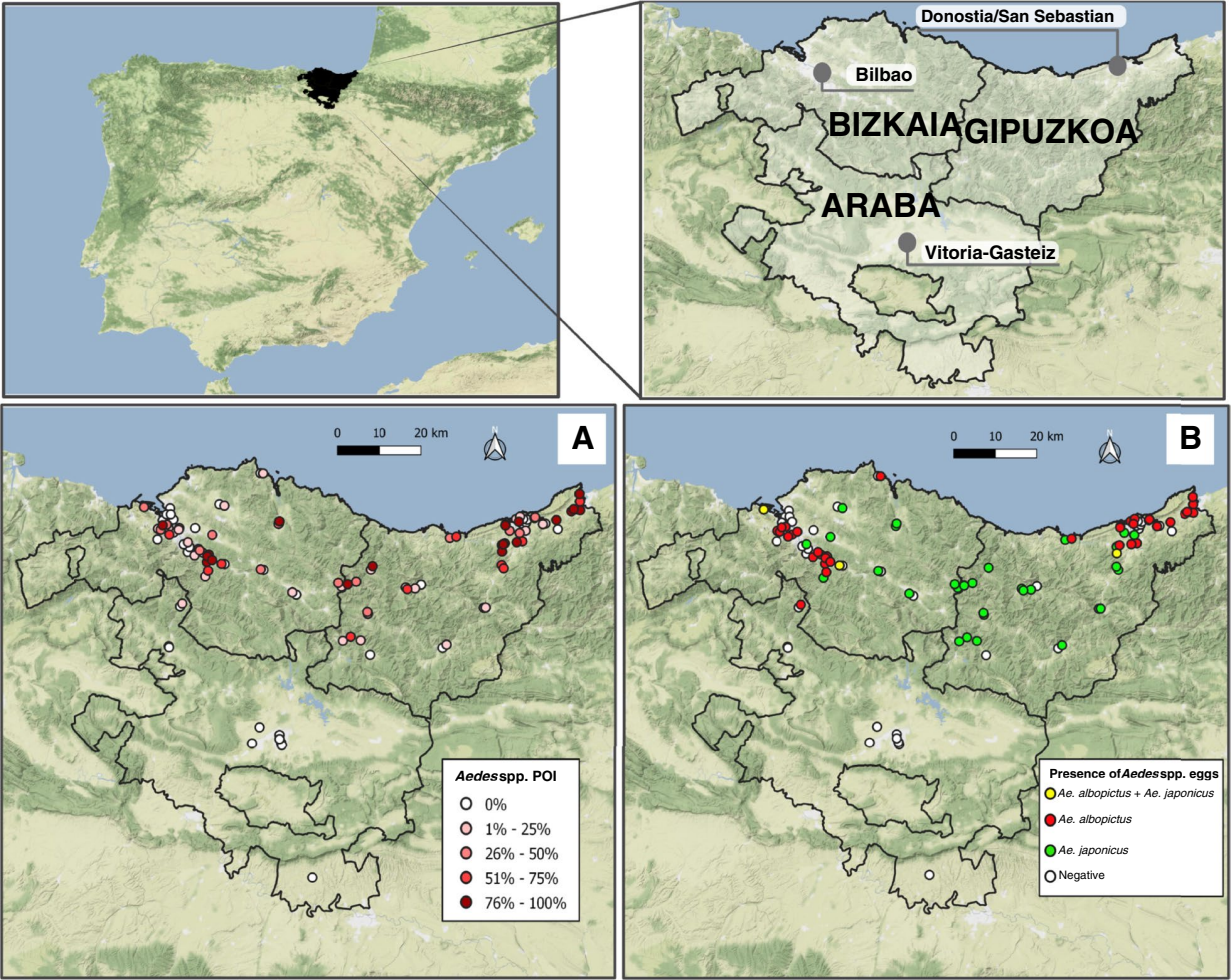
Distribution of *Aedes* spp. eggs in each sampling site according to the POI (Positive Ovitrap Index), represented in a gradient of color (**A**). Distribution of *Aedes albopictus* (red dots), *Ae. japonicus* (green dots), and coexistence of both species (yellow dots) (**B**)

The establishment of *Aedes* spp. according to POI was confirmed in several municipalities (Fig. 1a). The most intense red color indicates that between 75 and 100% of the ovitraps of the municipality were positive for the presence of *Aedes* eggs.

### Identification of *Aedes* spp.

To identify the species of *Aedes* in each sampling site, a total of 195 oviposition sticks with eggs were submitted to molting to obtain adult mosquitoes that were identified by morphological keys, as described above. When adult emergence failed, PCRs of the eggs and sequencing were carried out. The species of *Aedes* was identified in 40.2% of the positive oviposition sticks (191/475, 69 from Bizkaia, 120 from Gipuzkoa, 2 from Araba), confirming the presence of *Ae. albopictus* in 44 sampling areas from 23 municipalities, and *Ae. japonicus* in 39 sampling areas from 26 municipalities (Table 2; Fig. 1b).

*Aedes albopictus* was concentrated around large urban centers such as Bilbao and Donostia/San Sebastian, where the population density is higher and industrialized areas are abundant in the surrounding areas. *Aedes japonicus* predominated in less populated areas (Fig. 1b). Concurrence of both species was observed in 11 municipalities (Table 2). Both *Ae. japonicus* and *Ae. albopictus* were simultaneously present on the same oviposition stick on just one occasion. Eggs from *Aedes geniculatus* (Olivier, 1971) were identified by molecular methods in only one oviposition stick. No other *Aedes* species were identified by morphological or molecular methods.

### Oviposition activity of *Aedes* spp.

**Fig. 2:**
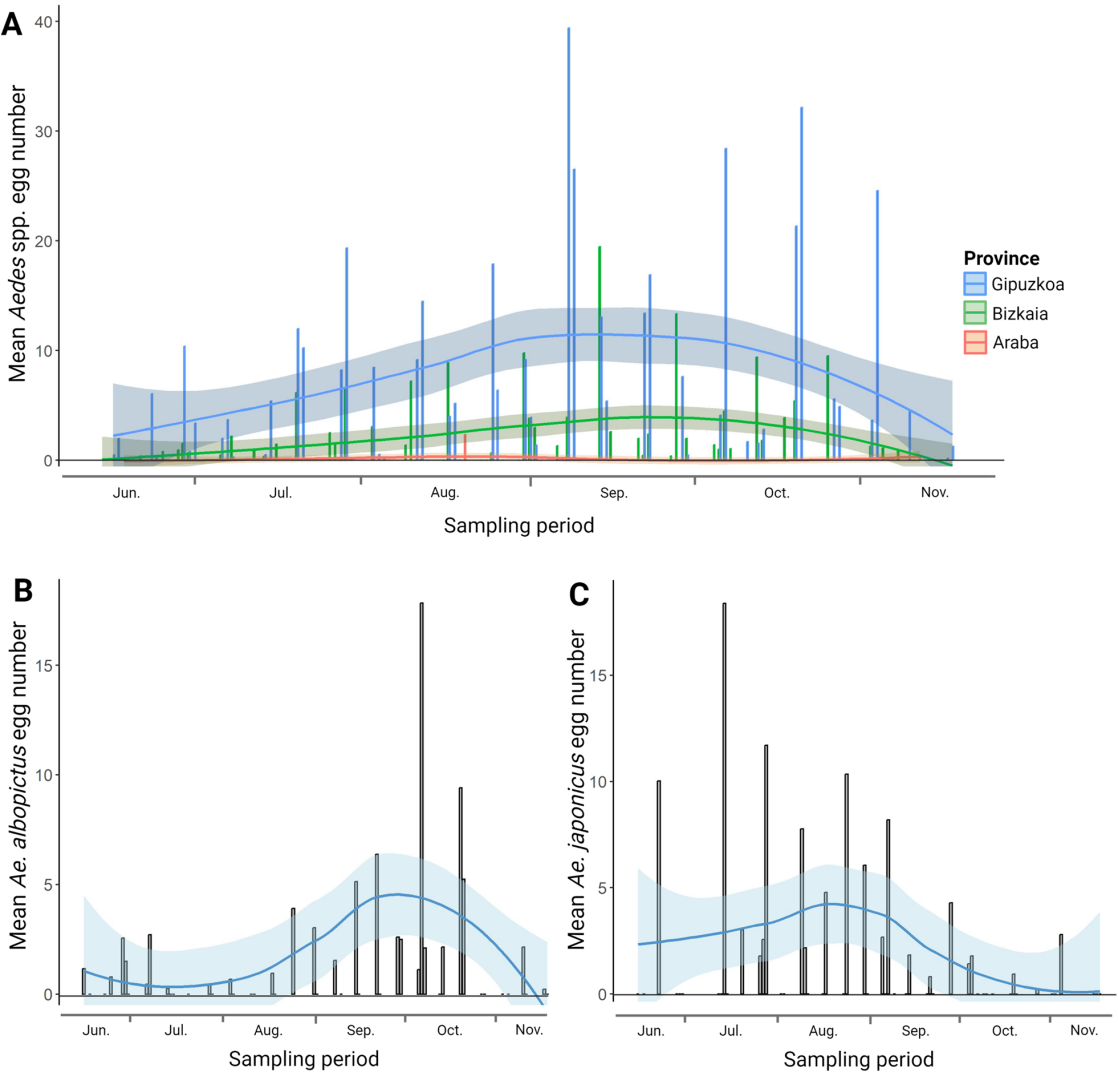
Mean *Aedes* spp. egg laying in the three Basque provinces (**A**). *Aedes albopictus* (**B**) and *Ae. japonicus* egg laying (**C**) considering only the municipalities where each species was exclusively found (12 and 15, respectively)

*Aedes* egg-laying activity progressively increased throughout the sampling period, with a maximum between September and November. *Aedes* egg laying was significantly higher in the province of Gipuzkoa than in Bizkaia and Araba (Kruskal-Wallis *χ*^2^ = 230.02, *P* < 0.0001) (Fig. 2a). Considering the 12 municipalities where only *Ae. albopictus* was identified, egg laying peaked between September and October (Fig. 2b). Similarly, considering the 15 municipalities with the only detection of *Ae. japonicus*, egg-laying activity showed a more stable pattern throughout the study period, with an earlier peak in July (Fig. 2c).

### Distribution of AIM by urbanization degree and characteristics of the environment

**Fig. 3:**
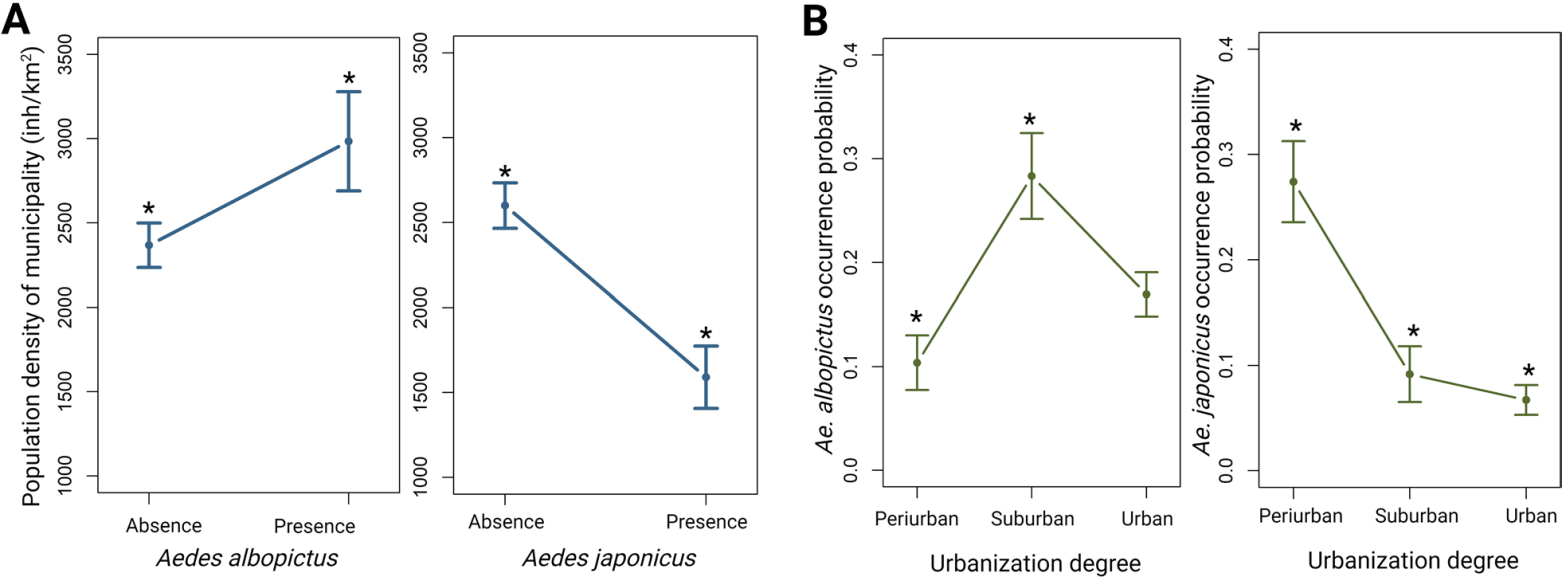
Presence of each species according to the population density (inh/km^2^) (**A**); occurrence probability of *Aedes albopictus* and *Ae. japonicus* according to the urbanization gradient (**B**)

The probability of *Ae. albopictus* occurrence was higher in Bizkaia (OR = 14.62) and Gipuzkoa (OR = 18.36) than in Araba, while that for *Ae. japonicus* was significantly high in Gipuzkoa (OR = 11.40) (Table 3). Interestingly, the presence of *Ae. albopictus* was associated with municipalities with a higher population density (mean = 2983 inh/km^2^) (*P* < 0.001), and the presence of *Ae. japonicus* was associated with lower population density (mean = 1590 inh/km^2^) (*P* < 0.05) (Fig. 3).

**Table 3:** Summary of logistic regression models for *Aedes albopictus* and *Ae. japonicus* regarding province, urbanization degree, and type of environment

Variables	*Aedes albopictus*			*Aedes japonicus*		
	*E* ± SE^a^	OR^b^ (95% CI)^c^	*P* value	*E* ± SE	OR (95% CI)	*P* value
Province						
Araba	Ref.^d^			Ref.		
Bizkaia	2.68 ± 1.02	14.62 (3.01–263.69)	0.009*	1.42 ± 1.06	4.15 (0.77–77.33)	0.180
Gipuzkoa	2.91 ± 1.02	18.36 (3.82–330.28)	0.004*	2.43 ± 1.03	11.40 (2.30–207.13)	0.018*
Urbanization degree						
Periurban	Ref.			Ref.		
Suburban	1.48 ± 0.35	4.39 (2.22–9.11)	0.001*	− 1.24 ± 0.39	0.28 (0.12–0.59)	0.001*
Urban	0.95 ± 0.36	2.60 (1.30–5.48)	0.008*	− 1.18 ± 0.36	0.30 (0.14–0.62)	0.001*
Type of environment						
Green park	Ref.			Ref.		
Petrol station/industrial area	0.42 ± 0.44	1.53 (0.63–3.65)	0.33	0.93 ± 0.46	2.55 (1.047–6.46)	0.041*
Housing	0.02 ± 0.30	1.02 (0.56–1.86)	0.94	− 0.06 ± 0.41	0.93 (0.40–2.13)	0.86
Parking	0.61 ± 0.30	1.84 (1.01–3.39)	0.047*	0.32 ± 0.41	1.38 (0.61–3.15)	0.42

Asterisk denotes *P *< 0.05

^a^Est ± SE = estimate ± standard error

^b^OR = Odds ratio

^c^95% CI = 95% Confidence intervals

^d^Ref. = reference category

The analysis between the presence of AIM and the degree of urbanization (urban, suburban, peri-urban) revealed that, whereas *Ae. albopictus* showed a 4.39 times higher probability of being found in suburban areas (*P* = 0.001) than in peri-urban areas, *Ae. japonicus* had a higher probability of being found in peri-urban areas than in suburban or urban areas (*P* = 0.001) (Table 3; Fig. 3).

Considering the characteristics of the environment, the distribution of both species relied on the type of site. Thus, the probability of finding *Ae. albopictus* was significantly higher in areas surrounding parking lots (*P* < 0.05), while the probability of finding *Ae. japonicus* was higher in areas surrounding petrol stations and industrial areas (*P* < 0.05) (Table 3).

## Discussion

Mosquitoes are of public health relevance when they populate in high densities, which causes a nuisance, or when they transmit pathogens. The successful establishment of invasive mosquito populations in new environments is a complex process influenced by several factors, including climate, habitat, and human activity [1]. Six invasive species have been established in Europe [2, 7], and among them, *Ae. albopictus* and *Ae. japonicus* are becoming widespread [7, 18]. The wide distribution observed in the Basque Country (northern Spain) confirms the spread and establishment of these species of invasive mosquitoes in the region. Our results demonstrate that *Ae. albopictus* predominated around the two main cities (Bilbao and San Sebastián) that harbor a larger population per km^2^ and more traffic and important industrial ring roads, which favor the introduction and distribution of this species [33, 34]. The province of Gipuzkoa was the probable point of entry of *Ae. albopictus* in the Basque Country [15], and since then, significantly higher activity has been detected in this province, as shown in the current study. Until recently, the AIM surveillance program in this region was focused on placing ovitraps in areas with high concentrations of traffic, which favored the detection of *Ae. albopictus* [15]. In 2020, the surveillance was extended to municipalities that were less populated and less industrialized, enabling the detection of *Ae. japonicus* in four municipalities [2]. Furthermore, the sampling strategy carried out in the present study (2021), which encompassed urban, suburban, and peri-urban areas, showed a wide distribution of *Ae. japonicus* in the studied region. It is difficult to know how long this species has been part of the entomofauna of the Basque Country and the possible route of entry. However, in the 2 years since its first detection [24], established populations of *Ae. japonicus* have been detected in a higher number of municipalities than *Ae. albopictus*.

In contrast to *Ae. albopictus*, which is distributed by passive dispersal through motorized vehicles, *Ae. japonicus* seems to be distributed over time by active dispersal [35], with an estimation of spreading more than 100 km within a few years [19, 22]. Thus, in countries such as Hungary or Italy, *Ae. japonicus* spread rapidly in a short period by appropriate ecological corridors, finding natural and artificial containers to breed [22, 36]. Considering that in our study the occurrence probability of *Ae. japonicus* is greater in Gipuzkoa (province bordering France), it could be speculated that this species may have come from France. However*, Ae. japonicus* seem to be established only in northeastern France [3], and there are no reports in southern France bordering our study area. It is important to highlight that, as this study demonstrates, if AIM surveillance is focused only on urban areas, the presence and distribution of *Ae. japonicus* may be underestimated.

Climatic and demographic variables, such as temperature, precipitation, and population density, are key factors in mosquito distribution [37]. In this study, a widespread presence of both species was observed in northern regions with a mesothermal Atlantic climate (moderate temperatures and extensive rainfall); however, no specimens were detected in the southern area with a transitional climate toward the Mediterranean (drier and warmer summers). *Aedes albopictus* has strong ecological plasticity, can be established in a wide range of different habitats with different climatic conditions, and is better adapted to warmer climates than *Ae. japonicus* [7]. These could be the reasons for the establishment of *Ae. japonicus* in regions of Spain with temperate Atlantic climates, such as northern Spain [24]. In addition, larvae of *Ae. japonicus* do not tolerate water temperatures > 30 °C [38], which occur during the summer in central and southern Spain.

In northern Italy, which enjoys colder winters and snow and relatively warm summers, the seasonal activity period of *Ae. japonicus* lasts at least 7 months between April and November [22]. However, in the study area, with milder winters, larvae were found in February and March in human-made containers around farmhouses in the provinces of Bizkaia and Gipuzkoa (unpublished data). This study also indicates that egg laying starts earlier for *Ae. japonicus* than *Ae. albopictus*. In fact, the predictions of species distribution according to the habitat suitability under climate change are different for both AIM species; whereas *Ae. albopictus* is promoted by climate change, the area modeled to be climatically suitable for *Ae. japonicus* is projected to decrease in Europe [39] as it would not be able to adapt to warmer climatic conditions.

In addition, landscape structure is key to facilitating the occurrence of *Ae. japonicus,* even in a climatically unsuitable region, and vice versa [40]. In general, the presence of *Ae. japonicus* is more prevalent in vegetation-rich [17, 41] and rural areas compared to urban and suburban areas [25] or in the transition zones between forest and settlements [42]. This study also showed a higher probability of finding *Ae. japonicus* in petrol stations and industrial areas. Both locations are usually surrounded by shrubs and vegetation-rich areas. Moreover, these areas offer optimal resting places for mosquitos in addition to a wide variety of artificial breeding containers. In contrast, the probability of finding *Ae. albopictus* has been higher in parking lots, which may be due to the role of terrestrial vehicles enabling the passive dispersion of this species [43, 44].

Urbanization processes modify the environment, have a major impact on the mosquito species community, and lead to biodiversity loss caused by anthropogenic changes [45]. The more urbanized a given area is, the fewer species are found, but mosquito species adapted to urban environments increase in abundance [46]. This is especially applicable to container-breeding and invasive mosquitoes [47, 48]. In the current study, the presence of *Ae. albopictus* and *Ae. japonicus* varied depending on the degree of urbanization. Thus, *Ae. albopictus* appeared concentrated in urban and suburban areas, probably because of the higher availability and density of artificial breeding sites, its tolerance to hotter and drier climate conditions, and the presence of a wider blood-feeding host range, including humans [49, 50]. In fact, this mosquito species is considered strongly anthropophilic, and in urban areas where the human population is greater, a higher blood-feeding rate has been observed [51]. In this study, *Ae. albopictus* showed a higher presence in municipalities with higher population densities. In contrast, *Ae. japonicus* showed a preference for municipalities with lower population density, such as peri-urban environments and rural settings [50]. Urban areas are occasionally colonized by *Ae. japonicus*, but hotter and drier summer conditions caused by the effect known as ‘urban heat island” [52] would negatively impact its life cycle. In addition, a higher variety of available mammal hosts to feed on could be the reason that they prefer peri-urban and rural settings [50]. Although the two species seem to coexist without much evidence of displacement, the potential competitive interaction between the larval stages of the two species should not be ignored and should be further investigated.

## Conclusions

This study confirms a new colonization area in Europe for *Ae. japonicus*. Even though *Ae. japonicus* is not considered a high-risk mosquito for public health, it is unknown whether the role of the species could change if its distribution and abundance increase. In addition, *Ae. albopictus* is expected to be fully established in the coming years, causing nuisance [15] and increasing the likelihood of autochthonous arbovirus transmission. The present results may have practical applications for the design of AIM surveillance. Due to the potential impact on public health and according to our findings, surveillance programs should be designed considering different types of environments, including municipalities with low population density and peri-urban areas. Mosquito surveillance and control activities will continue in the study area to keep population density at minimum. Special attention will be paid on the characterization of the *Aedes* breeding sites to better understand the distribution pattern of these two species of invasive mosquitoes.

## Data Availability

All data generated and analysed during this study are included in this published article.
